# Thermodynamic analysis of the thermal and exergetic performance of a mixed gas-steam aero derivative gas turbine engine for power generation

**DOI:** 10.1016/j.heliyon.2023.e18927

**Published:** 2023-08-03

**Authors:** J.B.W. Kok, E.A. Haselhoff

**Affiliations:** University of Twente, Laboratory of Thermal Engineering, PO Box 217, 7500AE, Enschede, the Netherlands

**Keywords:** STIG cycle, Steam injection, Aero derivative gas turbine, Mixed gas-steam cycle

## Abstract

A thermodynamic analysis is performed for an aero derivative gas turbine engine which utilizes steam injection to increase its efficiency. The target was to explore the performance of a high efficiency gas turbine unit for electric power generation without downstream Rankine cycle. A Rankine cycle for exhaust heat recovery is unattractive because of its large response time and cost of investment. The main purpose of this research was to develop a better understanding of how the optimal cycle efficiency is reached, when the steam for injection is generated by use of the turbine exhaust heat. The STIG cycle becomes attractive for grid stabilization because of its low CAPEX and small footprint and response time.

A thermodynamic model has been developed to simulate the simple cycle gas turbine, steam generation and effects of steam injection. Reference input parameters for the model are taken for the GE LM6000 turbine as publicly available. The performance of the engine without steam injection as predicted by the model is compared with literature for validation and compares well.

The performance of the STIG cycle as a function of operation parameter steam mass flow and design parameters pressure ratio and turbine inlet temperature is investigated and the optimal parameter settings determined. It is found that this type of cycle shows a very specific parameter setting for optimal efficiency. By using steam injection for the chosen turbine and its parameters an efficiency gain of around 11% points and an output power augmentation of 45% can be achieved.

## 1 Introduction

### 1.1 Background

Before the introduction of large scale solar and wind electric power generation, in Europe gas fired power generation plants were used the entire day in daytime in base load, to produce the power needed to meet demand. Nowadays a significant part of this power is generated by sustainable energy sources like solar and wind, and these have priority. Unfortunately, they are dependent on environmental conditions and therefore supply power in a highly variable and unpredictable manner. This would result in the destabilization of the electrical grid, if the variations could not be compensated by variable power on demand delivered by gas turbine power plants [1]. This is an issue from the operational point of view, but also from the view of return on investment, as the number of base load hours is reduced. The latter means that investments in steam-based Rankine cycles, as used in the combined cycle plants with their high CAPEX, become difficult.

The transient operation of a combined cycle plant is problematic since there is a high thermal inertia due to the Rankine cycle. This cycle takes hours to start-up rather than minutes and is therefore not suitable to be frequently interrupted. A simple cycle gas turbine (GT) however is much more flexible in transient operation and therefore very suitable for this purpose. Unfortunately, a GT has a much lower thermal efficiency as compared to the combined cycle. A method to improve the efficiency of a simple cycle GT is to recover part of the exhaust heat by means of a recuperator that preheats the compressor exit air. With the recuperator only the waste heat is recovered. The flue gas to air heat exchanger is however very large in volume and introduces additional pressure drop between compressor exit and combustor. With the transition to hydrogen as a fuel, preheating of compressor exit air becomes even less attractive with a view to risk of flashback in the combustor.

The GT simple cycle efficiency can be boosted alternatively by means of water/steam injection. Steam injection was used in the past to reduce pollutants like NO_x_ emission, but later it was noticed that with certain set-ups it could have additional performance benefits [2]. The steam is generated by recovery of the GT exhaust heat (STIG cycle). The STIG cycle not only recovers waste heat, but also reduces the compressor input power by reducing the compressor air flow by up to 15%. The latter is done at the cost of the pump power to the STIG water feed flow. The pump power is fortunately an order of magnitude smaller than the reduced compressor power. These two effects of the STIG cycle lead to higher efficiency than a recuperator. The objective of the present paper is to explore the performance improvement by using STIG, in view of the much faster response time as compared with a combined Brayton/Rankine cycle, smaller CAPEX, medium high efficiency and reduced NO emission. The latter becomes even more important for the transition of Natural Gas fired engines to hydrogen fired engines. This because hydrogen fired engines are more prone to nitric oxide formation, and the limiting effect of water vapor on this nitric oxide formation is probably necessary to operate within emission limits.

One of these set-ups was proposed in 1976 b y Prof. D. Y. Cheng, who showed that the heat of the GT exhaust gas could be used to generate high pressure steam in a heat recovery steam generator (HRSG). After injection of the steam in the GT this resulted in increase of efficiency and augmentation of power output. General Electric later used this technique on their aero-derivative engines and named them STeam Injected Gas (STIG) turbines [3]. Efficiency gains and power augmentation of 10% points and 50–70% respectively were shown to be achieved, however STIG turbines were not able to achieve the same maximum efficiencies as combined cycles, which reached up to 10–15% points higher values. As a result, the latter was preferred for base load power generation [4]. An additional drawback is their large high-quality water consumption, which is one of the reasons of its limited widespread use today [5]. Recovery of the steam by condensing the exhaust gas is therefore necessary to make it economically viable [[5], [6], [7]]. In view of the current transition to hydrogen as a fuel, opportunities for flue gas water recovery will grow however. A hydrogen fired engine will produce clean flue gas with residual oxygen and nitrogen and a relatively large fraction of water vapor, and no soot.

The STIG cycle is a very interesting and simple way to increase efficiency and augment power production for moderate plant sizes in the mid-power range of 1–50 MW. It was even shown that STIG performance can be better and specific work can be higher than the combined cycles under certain conditions [3]. It has the flexibility of a simple GT cycle and therefore can quickly adjust to the demand of power to stabilize the grid. Furthermore, implementing steam-gas cycles using existing aero-derivative gas turbine engines can be achieved without many modifications to the actual GT engine [8], which keeps the cost of investment low.

### 1.2 Objectives of research

The effects of STIG turbines in terms of efficiency increase, work augmentation and NOx reduction are well documented in literature. Novel research described in this paper is the analysis of how the most efficient injection point for STIG can be found. To provide more insight in the workings of the STIG an aero derivative GT will be selected for which the cycle will be analysed thermodynamically. A thermodynamic model is created for which the general parameters of design and operation are identified. The model results are compared to literature for validation and a performance comparison can be made with simple cycle performance. The main objective of this research is to make the cycle as efficient as possible by optimizing and matching the water injection with the exchanged heat of the exhaust gases in order to get an explanation how the most efficient point is reached. Target is to optimize the STIG cycle with a view to efficiency and power output as a function of the parameters mass flow of injection steam, pressure ratio and turbine inlet temperature. The study is performed on basis of heat input by combustion, and the fuel can be natural gas or hydrogen. The latter will become very important for application in sustainable power generation and very suitable to enhance the water recovery of the cycle.

## 2 .Theory

### 2.1 Gas turbine & STIG

**Fig. 1:**
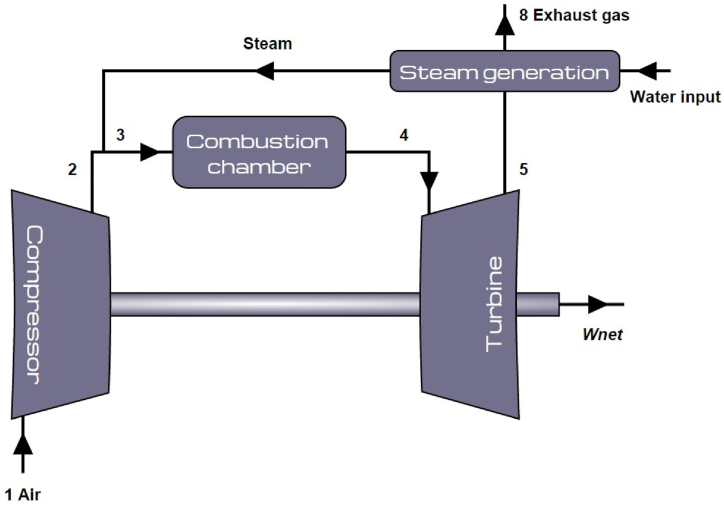
Schematic overview of the STIG cycle.

The steam injected gas turbine (STIG) is based on the idea that the high temperature exhaust gases can be used to generate high pressure steam, which is then injected into the gas turbine combustor. The core of the thermodynamic model built here consists of the simple Brayton cycle with isentropic efficiencies for compressor and turbine. This was expanded to the STIG set-up, for which a schematic can be seen in Fig. 1. Literature reports that the injected steam flow can amount to 10–20% of the air mass flow [5] and generation is limited by the amount of thermal energy available in the exhaust gas [9]. Both these points will be further analysed in section 4.

The turbine mass flow was kept at design point, even after steam injection in the model, as a result the airflow that enters the compressor is adjusted to account for the steam flow. This is technically possible with an aero-derivative engine thanks to the variable compressor inlet guide vanes. While this is beneficial for the back work ratio and the cycle efficiency, it also means that a mass-portion of steam now has to be heated by the combustion process to the turbine inlet temperature (TIT). Air has a specific heat of about 1 kJ/kg.K and steam 2 kJ/kg.K at 600 K ([10]). Because steam has a higher specific heat capacity than air, the amount of injected fuel will have to increase as well to ensure the same TIT.

As shown in Fig. 1 the steam injection takes place upstream the combustion chamber, the reasons being as follows. GTs have undesirable combustion products like NO_x_ formed due to high primary zone temperatures in the combustion chamber. When part of the combustion air is replaced by steam, the NO_x_ production is reduced up to a factor of 4 at unchanged adiabatic flame temperature [11]. This is due to the decrease of *O*_2_ in the oxidizer and due to suppression of *O*-radicals in the flame by the *H*_2_*O*. In view of the transition from Natural Gas fired to hydrogen fired gas turbine engines, the effect of NO_x_ suppression by steam might be essential for acceptable low NO_x_ emissions. Additionally, to prevent local high temperature spots the steam should be injected far up-stream of the Combustion Chamber to allow for proper mixing of the steam with the air [6,9,12]. Dilution of the oxidizer air by blending with steam downstream the compressor will lead to lower oxygen concentration and the steam will chemically suppress the nitric oxide formation as well. The influence of steam injection on the enthalpy, work and exergy transport in the gas turbine engine will be analysed according to the following basic equations for every point in the cycle [6,10]:

For the exergy atmospheric pressure and 288 K were taken as reference conditions. On basis of equations (1) to (5) a set of balance equations was derived over every component in the cycle. Solving these in the EES code allowed the computation of the net cycle output power and exergetic efficiency. In section 5 the exergetic performance of the turbine component will be analysed in more detail as a function of the steamfraction of the turbine flow, using the following equations:

(1) $$\text{First}\;\text{law}\;\text{of}\;\text{thermodynamic:}-\sum_{i=1}^{N}h_{i}m_{i}=Q-W_{shaft}$$

(2) $$\text{Compressor}:\eta_{isentropic}=\frac{\Delta h_{isentropic}}{\Delta h_{actual}}$$

(3) $$\text{Tubrine}:\eta_{isentropic}=\frac{\Delta h_{actual}}{\Delta h_{isentropic}}$$

(4) $$\text{Cycle}\;\text{thermal}\;{\;}_{\eta_{\text{th}}=\frac{W_{turbine}-W_{compressor}}{Q_{combustor}}}\;\text{efficiency:}$$

(5) $$\text{Exergy:}\;\psi_{i}=m_{i}\left(\left(h_{i}-h_{0}\right)-T_{0}\left(s_{i}-s_{0}\right)\right)$$

Specific turbine exergy change for mass component i (i being flue gas or steam):

(6) $$\psi_{turbine,i}=\left(h_{4,i}-h_{5,i}\right)-T_{0}\cdot \left(s_{4,i}-s_{5,i}\right)\left[kJ/kg\right]$$

Reversible turbine work for total mass flow:

(7) $$W_{rev}=m_{flue}\cdot \psi_{turbine,flue}+m_{steam}\cdot \psi_{turbine,steam}\left[kW\right]$$

### 2.2 Steam generation

For the generation of high-pressure steam, a HRSG is normally used. It has multiple sections in order to pre-heat/economize, evaporate and superheat the water, and this does have several drawbacks. The most important being that a cold start-up can take up to 2 h or more, which is caused mainly by the steam drum. As stated in the introduction, utilizing STIG on a turbine could prove to be a good solution for quick response power generation, thereby making the HRSG an unattractive addition. A very appealing alternative is the Once-Through Steam Generator (OTSG). The OTSG configuration is much simpler, exposing the exhaust gas to single pass tubes which run internally. The water is heated to steam in one run though the system, which therefore does not have loop sections like the HRSG. This allows for a much smaller thermal inertia that can achieve useable superheated steam in 15–30 min [13,14], which is much more desirable. The design of steam generators is not in the scope of this paper, but elaborately discussed in Refs. [[15], [16], [17]] for combined cycle coal and gas fired plants.

**Fig. 2:**
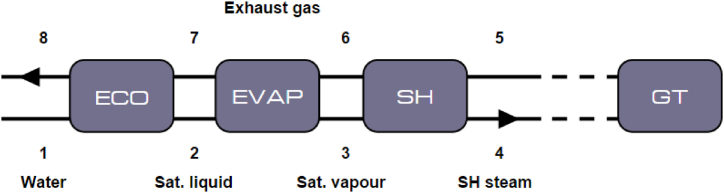
Schematic overview of the OTSG.

Since the process of economization, evaporation and super heating occurs in one pass, the mass-flow of water through the OTSG will therefore determine where these three specific processes will occur. Since the temperature of the exhaust gas at the inlet remains constant, this system is ideally designed to work at a certain water mass-flow rate. To create a model for the OTSG the exchanged heat between the exhaust gas and the input water is analysed; Fig. 2 gives a schematic representation. The 3 sections economizer, evaporator and superheater are indicated with the flue gas and water/steam flows with their characteristic input and output points. An important design parameter is to make sure there is always a minimum temperature difference between point 2 and 7, the pinch point, to make sure that during the entire processes heat is being transferred from the gas to the water [6].

The water used for the production of the injected steam has to be “high quality” de-mineralized. This makes the process more complex, however it is necessary to protect against high-temperature corrosion of internal GT parts like the turbine blades [3]. This is especially important for aero-derivative engines, which need higher quality water compared to “heavy-duty” land engines [5]. In order to prevent excessive high-quality water consumption, the injected steam could be condensed and recovered from the exhaust gas in order to recycle it for steam generation. Literature shows that practical solutions could be available, however more research in this area for actual implementation of water vapor recovery is still possible [3,5,9]. The recent development to hydrogen fired gas turbine engines is an opportunity to supply mixed steam gas turbines relatively easy with high quality water. When hydrogen fired, the flue gas is relatively clean and composed of residual oxygen, nitrogen, a large amount of water vapor and no carbon dioxide nor soot. The water vapor is produced by the injected steam and by the combustion process. By adding a condenser to the exit, the hydrogen combustion based clean flue gas can be dried and the water consumed by the cycle can be recovered.

### 2.3 Engine choice and parameters

An aero-derivative GT is considered since this type of engine is attractive for several reasons. First of all if compared to industrial gas turbines they have a quick start-up capability and can do this frequently without causing significant maintenance costs [18]. Another very advantageous property is that the compressor has variable inlet guide vanes with which the intake of air can be reduced. This feature avoids a portion of the compressed air has to be bled in order for the GT to accept the injected water and maintain the designed turbine mass-flow. Reduction of the air intake to the compressor, while keeping turbine mass flow constant with steam injection, reduces compressor work and hence leads to efficiency gain [2]. Furthermore, the higher the PR of a GT, the higher its efficiency is. Aero-derivative engines have very high PRs - around 30 or more-therefore reach high simple cycle efficiency values of around 40% [5]. It could also be considered to design a new type of GT specifically created for mixed gas-steam usage. While this could certainly be achieved, considering the R&D costs related to creating a new engine and its employment being very specific, this option is unattractive. Moreover, an aero-derivative engine only needs minor adjustments to implement STIG, which makes it economically a more feasible option because of its low cost of investment [8]. Reference [19] developed a model similar to the model in the present paper to analyse the performance of given STIG and intercooled cycles, but did not aim at optimizing the steam flow as a function pressure ratio and turbine inlet temperature. Their results show that intercooling and steam injection are both sensible applications for gas turbines especially when both of them are considered. They confirm that advantages such as high thermal efficiency, low NOx emissions, and simple design are realized more extensively, and expect that STIG cycles would be exploited more in future gas turbine applications. Reference [20] investigated a STIG cycle, to which they referred as TopCycle and made comparisons to a combined cycle plant. The cycle configuration is presented in detail, and its operation is exemplified on the basis of simulation results for specific operational points. They conclude tha operation at design condition results in electric efficiencies higher than 50% (lower heating value (LHV)). The TopCycle operates at an elevated electric efficiency and considerably higher power density, which can be transferred into smaller plant footprint and dimensions and thus lower investment costs at equal power output in comparison to a CC plant.

The target of the present study is to analyse how to optimize a high efficiency gas turbine engine in simple cycle operation with improved performance by steam injection as a function of the parameters steam mass flow, pressure ratio and turbine inlet temperature. The steam is generated on basis of exhaust heat, thereby recovering high temperature energy. In order to introduce steam into the exit of the compressor, the compressor air flow needs to be decreased to keep the turbine inlet temperature at design point. This is possible by adjusting the Inlet Guide Vanes with an aero-derivative engine and in addition the aero-derivative has a higher simple cycle efficiency as compared to a landbased design. General Electric produces several types of these GTs. At the time of writing their latest models are the LM6000 series, which generate power between 40 and 50 MW. They have PRs that reach over 30 and efficiencies of around 42% [21]. The LM6000-PG was chosen because it is representative for an aero-derivative engine, its operational details are relatively well available in the literature and it has one of the highest PRs. Input parameters for the model are taken from data sheets as provided by General Electric [21,22], which are presented in Table 1.

**Table 1:** Manufacturer data LM6000-PG.

^*T*^1,*amb*	15	◦_C_
^*W*^*net*	52,4	*MW*
^*P*^*ratio*	33,2	
Exhaust flow	141	*kg*/*s*
Exhaust *T*	499	◦_C_

## 3 Model

The analysis of the STIG is done with help of the thermodynamic program Engineering Equation Solver (EES), which is a software package used to find solutions for a system of algebraic equations including a thermodynamic properties database. In addition it contains many material properties, specialized functions and can solve iterative problems making it a powerful tool for solving thermodynamic and heat-transfer problems. The algebraic equations are set up in terms of balance equations for each component and each flow on basis of equations (1) to (7). More detailed descriptions can be found in Refs. [18,19]. In our case the set up was supported by the use of the EES solver and its thermodynamic database. The model consists of three parts: the simple GT, the addition of STIG, and the coupling to the generation of steam. The used EES code is made available at the repository of the Dutch 4TU. In the thermodynamic model some parameter values are necessary as an input, such as the TIT and compressor and turbine efficiencies that are not provided by the OEM. In addition to the manufacturer data as seen in Table 1, assumptions have been made for these parameters, which can be seen in Table 2. They are adjusted such that the model prediction for the simple GT model compares well to the OEM performance data. See Table 3 for a comparison from which can be determined that the simple GT model is able to closely predict the OEM specified power output and turbine outlet temperature while having a slightly higher efficiency compared to the chosen turbine. In fact for the exemplary character of the analysis the choice of parameter values is not very critical provided we use the same values throughout the paper.

**Table 2:** Model parameter assumptions.

TIT	1260	◦_C_
η_*c*_	93	%
η_*t*_	86	%
^*LHV*^*methane*	50,000	*kJ*/*kg*
^η^*cc*	100	%

**Table 3:** Manufacturer data compared with model results.

Parameter	Manufacturer	Model	Unit
^*P*^*ratio*	33,2	33,2	–
^*m*^*tot*	141	141	*kg*/*s*
^*T*^1;*amb*	15	15	◦_C_
^*W*^*net*	52,4	51,8	*MW*
^η^*cycle*	41,6	42,2	%
Heat rate	8660	8537	*kJ*/*kWh*
Exhaust T	499	495	◦_C_

## 4 Analysis of results

### 4.1 Verification with literature

**Fig. 3:**
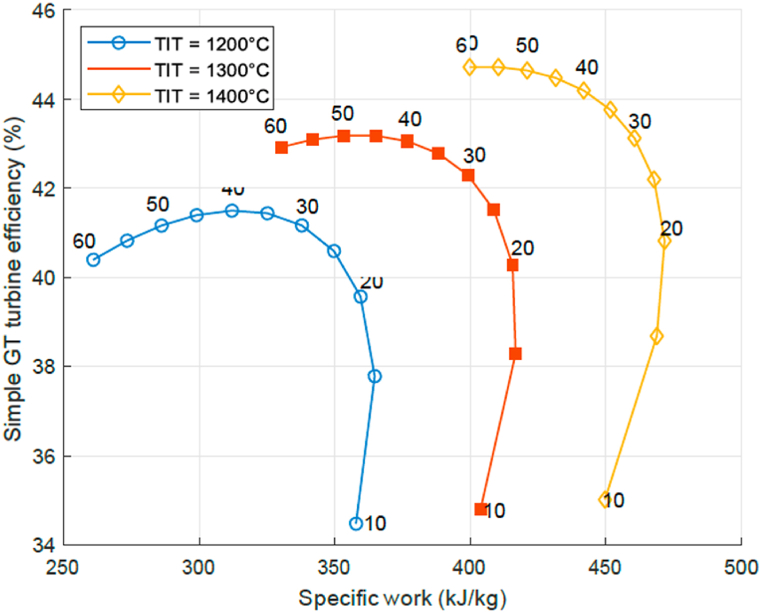
Simple cycle GT efficiency as a function of specific work for given P-ratios at *T IT* = 1200, 1300, 1400 °C.

Starting the analysis for the simple GT, the thermal efficiency as a function of the specific work at several PR and TIT can be computed. Results are shown for several TIT values with the PR indicated by a number in the curves in Fig. 3. Note that the PR-range goes from 10 to 60, and TIT-range from 1200 °C to 1400 °C; the bottom end of the range values being low, while the top end values are very high if compared to current standards. This is to explore the parameter sensitivity and to have an outlook to future technology. It can be observed in all 3 curves, that the efficiency is low at low PR, but the specific work output high. With increasing PR the specific work output decreases and the thermal efficiency increases. The thermal efficiency increases however to a maximum at a PR of about 30–40, depending on the TIT. For higher PR the efficiency is decreasing again, and so is the specific work. Moving to higher values of the TIT the optimum shifts to higher specific work, optimal PR, and efficiency values. This pattern is characteristic for GTs and is well known in literature [[5], [6], [7]]. For a given TIT there is a certain PR where the efficiency is at a maximum. Increasing the PR beyond the value where maximum efficiency is reached is therefore disadvantageous since both efficiency and specific work output decrease. Current turbines operate at a TIT around or above 1200 °C and PR values of around 30 [6], which means that efficiency benefits from a PR increase are still possible for a simple cycle GT for the assumed parameters.

**Fig. 4:**
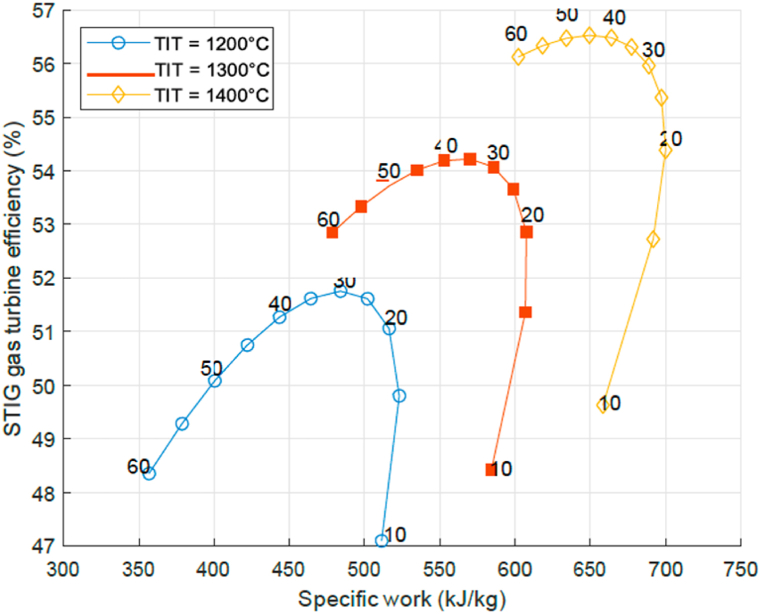
STIG efficiency as a function of specific work for given P-ratios at *T IT* = 1200, 1300, 1400 °C.

A similar analysis is performed for the STIG cycle. Now a certain steam fraction (x) is injected, defined as a fraction of the total mass flow through the turbine. For every PR value, the system utilizes the most efficient injection point. How the most efficient injection point is determined is described in section 4.2. Fig. 4 shows the computed thermal efficiency for the STIG cycle as a function of the specific work output with as a parameter the pressure ratio. A similar behaviour as observed in the simple cycle GT is observed on the STIG cycle for the relation between efficiency and specific work output. This is confirmed by literature [7]. Fig. 4 shows that in comparison with a simple cycle GT, a STIG cycle reaches much higher thermal efficiency and higher specific work output at a lower PR. For example, at a TIT of 1200 Celsius the STIG reaches 52% efficiency at 470 kJ/kg specific work at a PR of 30. These values are for a simple cycle GT respectively 41%, 320 kJ/kg and PR of 40. This maximum performance at a PR of 30 for a STIG cycle is a very advantageous characteristic, since this would mean that for the assumed parameters current turbines already have a suitable PR for maximum performance for STIG.

**Fig. 5:**
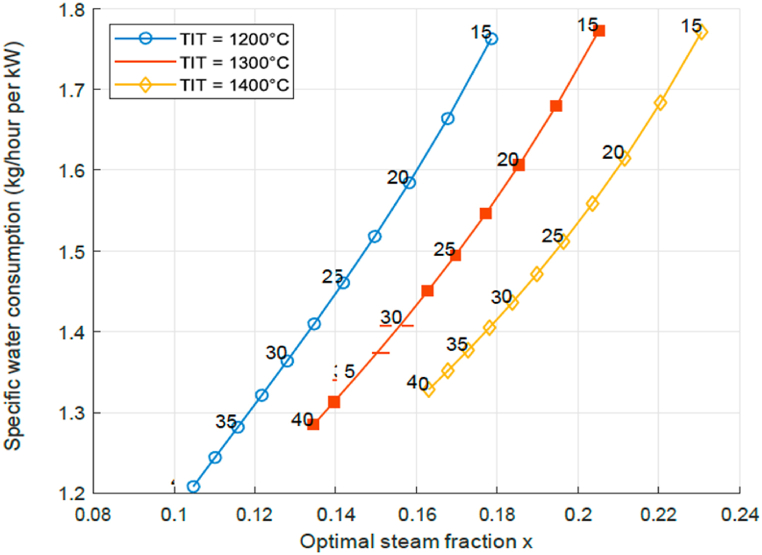
STIG specific water consumption and optimal steam fraction at P-ratios for *T IT* = 1200, 1300, 1400 °C.

Another important factor is the water consumption. For a given TIT and PR, the STIG cycle will have a specific optimal Steam Fraction *x* and specific water consumption (kg water/hour per kW). The relation between these two variables is shown in Fig. 5 as a function of 3 TIT values and with indication of the PR at each location of the curve. It can be seen that if the pressure ratio PR is increased at constant TIT, the specific water consumption will decrease. If the TIT is increased, so does the water specific consumption at constant PR. This observation is consistent with literature [7,23]. Notice that with Fig. 4, Fig. 5 one could determine the optimal pressure ratio PR and steam fraction SF for a given TIT, and then find the resulting efficiency, specific water consumption and work output.

### 4.2 STIG efficiency

**Fig. 6:**
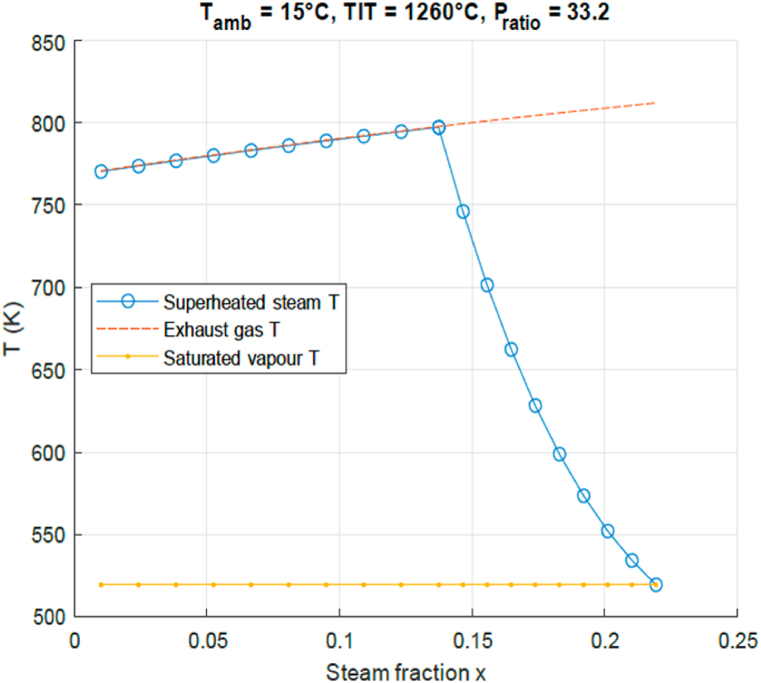
Highest possible steam temperature for steam mass-fraction SF.

As observed above, the STIG cycle has a very distinctive point at which it operates at the highest efficiency. In order to understand this, a number of characteristics of the STIG cycle are discussed. Therefore the diagrams 6 to 10 are generated by using the model-values as shown in Table 2, Table 3 Fig. 6 shows the highest possible steam temperature for a certain steam mass fraction SF, at given PR and TIT, in the last step in the creation of steam. Here the steam is superheated prior to injection into the combustor inlet. There are two important characteristic temperatures: the saturated vapor temperature of the water flow, and the turbine outlet temperature (red dotted line, for the heating of the water flow). For a given steam fraction *SF* the line marked with circles shows to which temperature the steam can be superheated. For example, at *x* = 0.17 the generated steam would be around *T* = 630*K*. If the SF is decreased, the result would be that the exhaust heat is transferred to a smaller mass-flow, resulting in a higher overall temperature. The opposite also happens. If the steam fraction is increased the exhaust heat is now transferred to larger mass-flow, resulting in a lower overall temperature. Notice that around *SF* = 0.22 it hits a limit. The mass-flow of steam has become so large that there is just enough heat to produce a saturated vapor. SFs higher than this specific point can therefore not be achieved by solely using the turbine exhaust gasses. Furthermore, the left side of the graph consists of relatively low SFs. As a result of this small steam mass-flow, there is more than enough heat in the turbine exhaust gas to bring it to the maximum temperature; its limit value being assumed to be equal to the exhaust gas temperature.

Three important regions can now be distinguished. Region I (0 < SF < 0.14) where the SF is sufficiently low, the steam can be brought up to a maximum temperature, region II (0.14 < SF < 0.22) where the steam can be heated to a certain temperature but lower than maximum, and region III (SF > 0.22) which consists of SFs which cannot be generated by solely using the turbine outlet gasses.

**Fig. 7:**
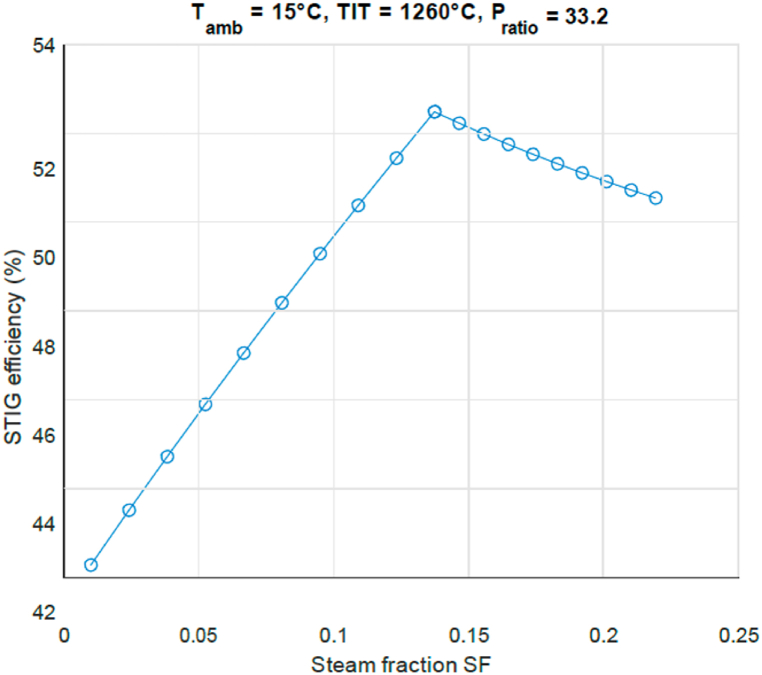
STIG cycle efficiency for amount of injected steam SF.

Fig. 7 shows the thermal efficiency as a function of steam mass fraction, for the conditions of Fig. 6. The STIG efficiency increases linearly with the steam fraction from 42% to an efficiency as high as 53% at steam mass fraction 0.14. This is confirmed by results for a different engine in Ref. [18]. In Fig. 7 it is observed that, where regions I and II meet, the most efficient point can be found at *x* = 0.14 and efficiency = 0.53. At this point the SF is such, that the cycle absorbs the maximum turbine exhaust heat for steam generation while still reaching the maximum temperature. On both sides of the optimum point the efficiency drops rapidly. A comment can be made on the possibility of moving to rich or stoichiometric combustion when air flow is replaced by steam flow. Near stoichiometric combustion must be avoided with a view to emission of nitric oxides and unburnt fuel. For the gas turbine combustor the air factor is defined as the ratio of the air flow supplied by the compressor to the air flow necessary for stoichiometric combustion. For low emission of harmful gases the air factor needs to exceed unity. Gas turbine combustors are operated at an air factor of about 2. In the paper it is shown in Fig. 7 that the maximum efficiency of the STIG cycle is reached at a steam volume fraction of 0.15. So in that case the air factor is reduced to 0.85 × 2 = 1.7, while keeping the combustor temperature constant. Hence there is still abundant oxygen for completed combustion and the emission of nitric oxides is even decreased due to the restraining effect of the H_2_O molecule on nitric oxide production.

**Fig. 8:**
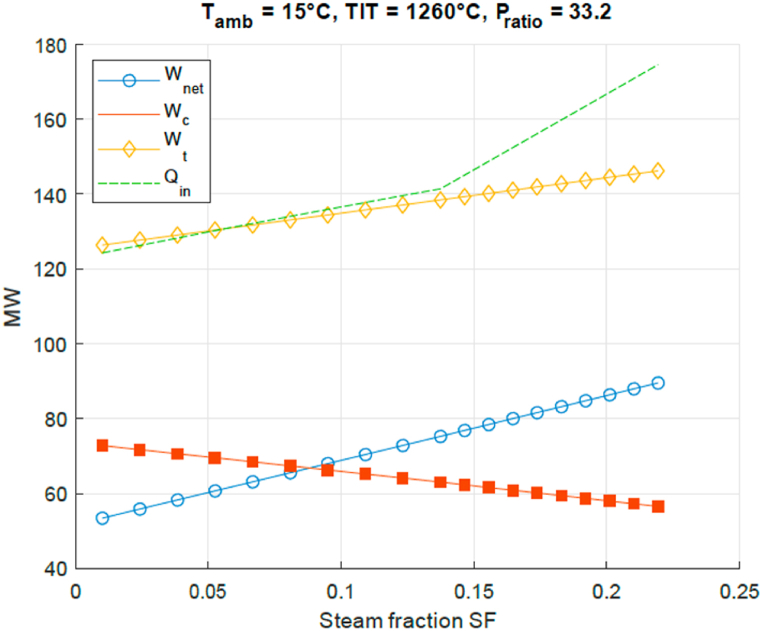
STIG cycle power output analysis for amount of injected steam SF.

In order to analyse the results shown in Fig. 4, Fig. 5, Fig. 6, Fig. 7, the processes that define the efficiency are shown in Fig. 8. Here as a function of steam fraction SF the blue circles line shows the net work output, consisting of the difference between the compressor work (red squares line) and the turbine work (yellow diamonds line). The fuel consumption is determined by the heat input as given by the green line. It can be seen that the higher the steam fraction, the less compressor work is needed, while the turbine output work increases; resulting in an increase of the net work output *W*_*net*_ produced by the GT. Also as previously stated, it can be seen that *Q*_*in*_ rises with higher steam injection since more fuel needs to be burnt to bring the steam to the same TIT. For region I the gain in *W*_*net*_ is higher than the increase of *Q*_*in*_, therefore it is beneficial for thermal efficiency to increase the injected steam fraction. In region II (steam fraction above 0.15) however the temperature of the steam drops rapidly if the steam fraction is increased, as seen in Fig. 7. This results in additional fuel that needs to be burnt to bridge that gap, which increases the total amount of needed *Q*_*in*_. In that situation it is beneficial to decrease the steam fraction to 0.15. This therefore results in an optimal point where these two regions meet.

**Fig. 9:**
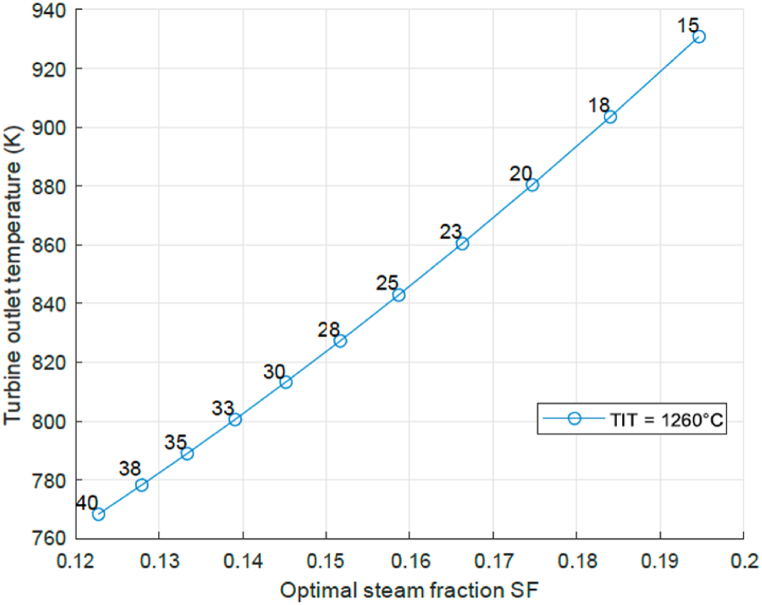
Turbine outlet temperature and optimal steam fraction SF for related pressure ratio at given TIT.

At given turbine inlet temperature the turbine outlet temperature will decrease with increasing pressure ratio, as the turbine gases are expanded over a larger pressure range and more work (i.e. enthalpy) is extracted. Lower exhaust gas specific enthalpy means a lower temperature ([10]). This has consequences for the steam generated downstream the turbine. Fig. 9 presents the relation between turbine outlet temperature and optimal steam fraction SF for given TIT. As to be expected, the optimal steam mass fraction SF decreases with decreasing turbine outlet temperature (and increasing PR). This figure demonstrates that in order to minimize water consumption the pressure ratio PR will have to be high and explains the relation between water consumption and optimal SF presented in Fig. 5. In order to find the optimal thermal efficiency, the turbine flow has to be maximized too and that was presented in Fig. 4. This demonstrates that the optimal efficiency is reached by means of maximization of turbine work in the balance between increasing specific turbine output and increasing turbine mass flow. A low PR engine is not advised as the required steam mass flow will be high.

## 5 Second law analysis

**Fig. 10:**
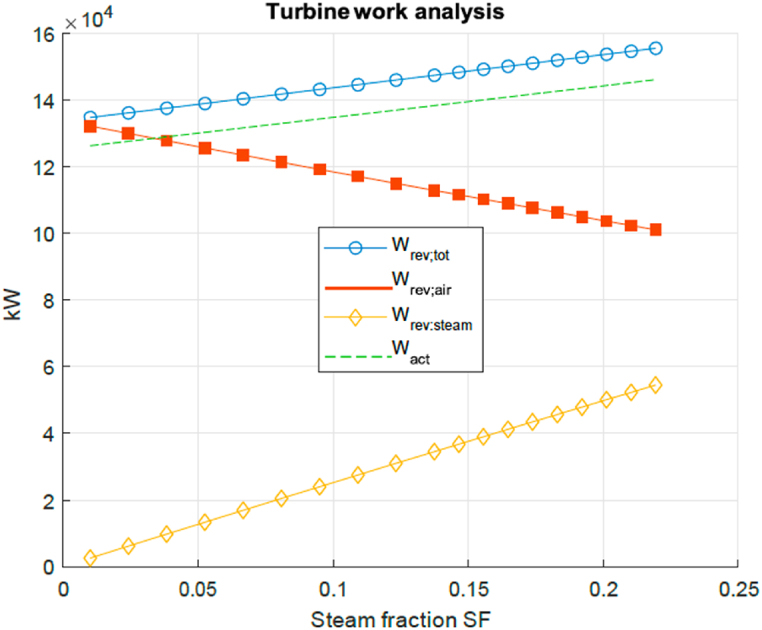
Turbine work analysis for certain steam injection SF.

**Fig. 11:**
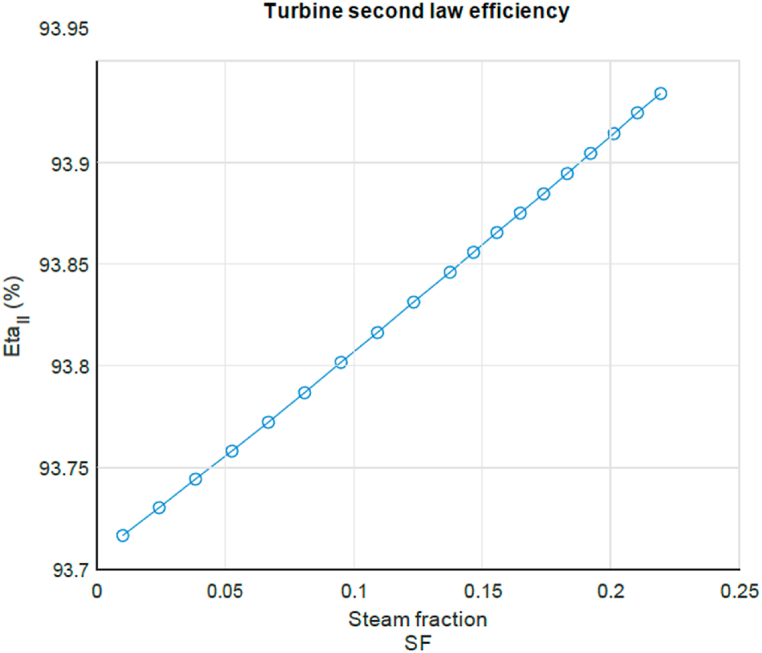
Second law turbine efficiency as a function of steam mass fraction.

Next a look is taken at the influence of the injection of steam for the cycle flow of exergy and the second law efficiency when the blend of flue gas and steam passes through the turbine. For this equations (6) and (7) are used. Fig. 10 presents a turbine work analysis for certain steam injection steam mass fraction SF. The blue line shows the total amount of reversible work (exergy) that could have been produced by the turbine by expanding the hot flue gas and steam. The reversible work contribution of the hot flue gas (red squares line) and steam flow (yellow diamonds line) components are shown as a function of steam fraction. As can be seen, the contribution of the reversible work of the hot flue gas reduces with half the amount that the contribution of the steam increases for a given increase in SF. This results in a continuous increase of the total reversible work with increase of the SF. The green line shows the actual work produced by the turbine, taking into account turbine efficiency. The green line has the same inclination as the blue line (total reversible turbine work) but at a smaller off-set due to the irreversible losses of the turbine. From this it can already be seen that the exergy loss remains approximately constant over the entire SF range. This is not a surprise, as the turbine inlet temperature, pressure and pressure ratio remain constant. When computing the second law exergetic efficiency this is indeed visible, see Fig. 11. It shows that there is a slight increase of around 0.2% points on the entire range, making the effect of steam injection therefore negligible in terms of turbine exergetic efficiency. This can be explained by the conversion of exergy in the exhaust gas to exergy in steam and subsequently increased work output by the turbine.

## 6 Conclusions and recommendations

From the analysed data a few conclusions can be drawn. First of all, when comparing the results from the models with various literature references, it is seen that the characteristic behaviour is present for the GT as well as the STIG. For example, GTs have a maximum PR for a certain TIT where the cycle efficiency is at its highest. The STIG shows similar behaviour, but for lower PR values, while achieving higher values for cycle efficiencies and specific work. Additionally, it is seen that increasing the TIT at a constant PR value increases the optimal specific water consumption, while increasing the pressure ratio at constant TIT reduces it. Hence using STIG leads to the highest increase in efficiency for a low PR engine, but also this low PR engine will have a lower generic efficiency when compared to a high PR engine. It is shown that the optimal PR is determined by the turbine inlet temperature, as expected, but it also determines the optimal steam flow rate. For modest water consumption and high efficiency a gas turbine engine with high pressure ratio is preferred. Hence the analysis confirms the hypothesis that aero-derivative engines are preferred over land power engines.

When optimizing the STIG it was found that the most efficient point can be reached for the Steam Fraction where the generated steam utilizes the maximum heat flow from the exhaust gasses while still reaching the maximum turbine inlet temperature, hence having the highest enthalpy before injection. In practice this point is reached around steam mass fraction 0.14.

At the most efficient design point, with the STIG cycle for the chosen turbine, an efficiency increase of 11% points and work augmentation of around 45% can be achieved. The effects of steam injection on the second law efficiency for the turbine section are negligible because of the high exhaust temperature of the simple GT.

Current aero derivative gas turbine engine designs have a pressure ratio of 33 and turbine inlet temperature of slightly over 1260° Celsius. This is already a very good operational environment for steam injection on basis of exhaust gas heat recovery with steam mass fraction 0.14 and efficiency increased to 52%. The developments for aero derivative engine designs might move to higher pressure ratio's and turbine inlet temperatures. If the pressure ratio becomes for example 45 and the turbine inlet temperature around 1330 Celsius, the steam mass fraction is increased to 0.16 and the efficiency to 55% on basis of Fig. 4, Fig. 5.

Additional research is suggested. The high-quality water consumed by the STIG needs to be recovered from the exhaust to decrease treatment costs. There has been research in this area [3,5,9]. This is in particular relevant with the current transition from natural gas to hydrogen powered gas turbine engines.

## Author contribution statement

J.B.W. Kok, E.A. Haselhoff: Conceived and designed the experiments; Performed the experiments; Analysed and interpreted the data; Contributed reagents, materials, analysis tools or data; Wrote the paper.

## Data availability statement

Data associated with this study has been deposited at https://data.4tu.nl/.

## Declaration of competing interest

The authors declare that they have no known competing financial interests or personal relationships that could have appeared to influence the work reported in this paper.

## Nomenclature

**Abbreviations**
CC: combustion chamber
EES: engineering equations solver
GT: gas turbine
OTSG: once through steam generation
PR: pressure ratio
SF: steam fraction
STIG: steam injected gas turbine engine
TIT: turbine inlet temperature

**Symbols Unit**
*c*: specific heat capacity *kJ*/*kgK*
η: efficiency - *h* enthalpy *kJ*/*kg*
*m*˙: mas-flow *kg*/*s*
ψ: exergy *kJ*/*kg*
*s*: entropy *kJ*/*kgK*
*W*^˙^: work *kJ*/*s*
*w*: specific work *kJ*/*kg*
*Q*^˙^: heat *kJ*/*s*
*q*: specific heat *kJ*/*kg*
*T*: temperature *K* or ^◦^C

**Subscripts**
*a*: air
*amb*: ambient condition
*eco*: economizer
*c*: compressor
*cc*: combustion chamber
f fuel
g combustion gas
*is*: isentropic
*p*: pressure
s steam
*sh*: superheated
t turbine
*vap*: evaporation
*w*: water
